# Intraoperative FLIP to guide revisional hiatal hernia repair: early experience and outcomes

**DOI:** 10.1007/s00464-026-13247-w

**Published:** 2026-08-24

**Authors:** Paul Symansky, Connor Caputo, Hemasat Alkhatib, Ramiro Cadena-Semanate, Voranaddha Vacharathit, Kevin El-Hayek

**Affiliations:** 1 Division of General Surgery, Department of Surgery, The MetroHealth System, 2500 MetroHealth Drive, Cleveland, OH 44109 USA; 2 Case Western Reserve University School of Medicine, 10900 Euclid Ave., Cleveland, OH 44106 USA; 3 Department of Surgery, Faculty of Medicine, Chulalongkorn University, Bangkok, Thailand; 4 King Chulalongkorn Memorial Hospital, The Thai Red Cross Society, Bangkok, Thailand

**Keywords:** Revisional hiatal hernia repair, Functional lumen imaging probe, EndoFLIP, Foregut surgery, Gastroesophageal reflux disease, Distensibility index

## Abstract

**Background:**

Revisional hiatal hernia repair (rHHR) represents a challenge in foregut surgery, given scarring, distorted anatomy, and high risk of morbidity. Traditional methods of intraoperative assessment, such as bougie calibration and endoscopic inspection, are subjective and lack physiologic correlation. The functional luminal imaging probe (FLIP) offers objective, real-time assessment of esophagogastric junction (EGJ) diameter, distensibility, and cross-sectional area. This study evaluates the utility of intraoperative FLIP to guide surgical decision-making and assess outcomes in a cohort of complex patients undergoing rHHR.

**Methods:**

A retrospective review was conducted evaluating patients who underwent FLIP-guided rHHR from 2020 to 2025 at an urban safety-net hospital. Intraoperative FLIP measurements, specifically the diameter (D) and distensibility index (DI), were used to tailor surgical management, including decisions to take down, preserve, or alter the fundoplication. Longitudinal follow-up assessed symptom resolution and quality of life (QOL) using the GERD-Health-Related Quality of Life (GERD-HRQL) questionnaire.

**Results:**

A total of 17 patients undergoing 18 revisional operations were analyzed (mean age 47.5 years; 88% female). Intraoperative FLIP values prompted a change in surgical management in 61% (11/18) of cases. Modifications included preservation of prior fundoplication (36%), tightening an existing fundoplication (27%), deferral of fundoplication (27%), and changing fundoplication type (9%). At a mean follow-up of 24.9 months, GERD-HRQL scores improved from 26.7 ± 8.5 preoperatively to 16.5 ± 12.9 postoperatively (*p* = 0.034). At long-term follow-up (mean 38.8 months), persistent symptoms were noted, including reflux requiring medication (70.6%) and dysphagia (35.3%), though 64.7% of symptomatic patients were managed nonoperatively.

**Conclusions:**

FLIP-guided revisional HHR facilitates a tailored surgical approach, prompting alterations in management based on objective physiologic data. While clinically meaningful improvements in quality of life were observed, the persistence of symptoms in this high-risk population highlights the intrinsic complexity of revisional foregut surgery and the need for future prospective studies.

**Graphical Abstract:**

**Figure:**
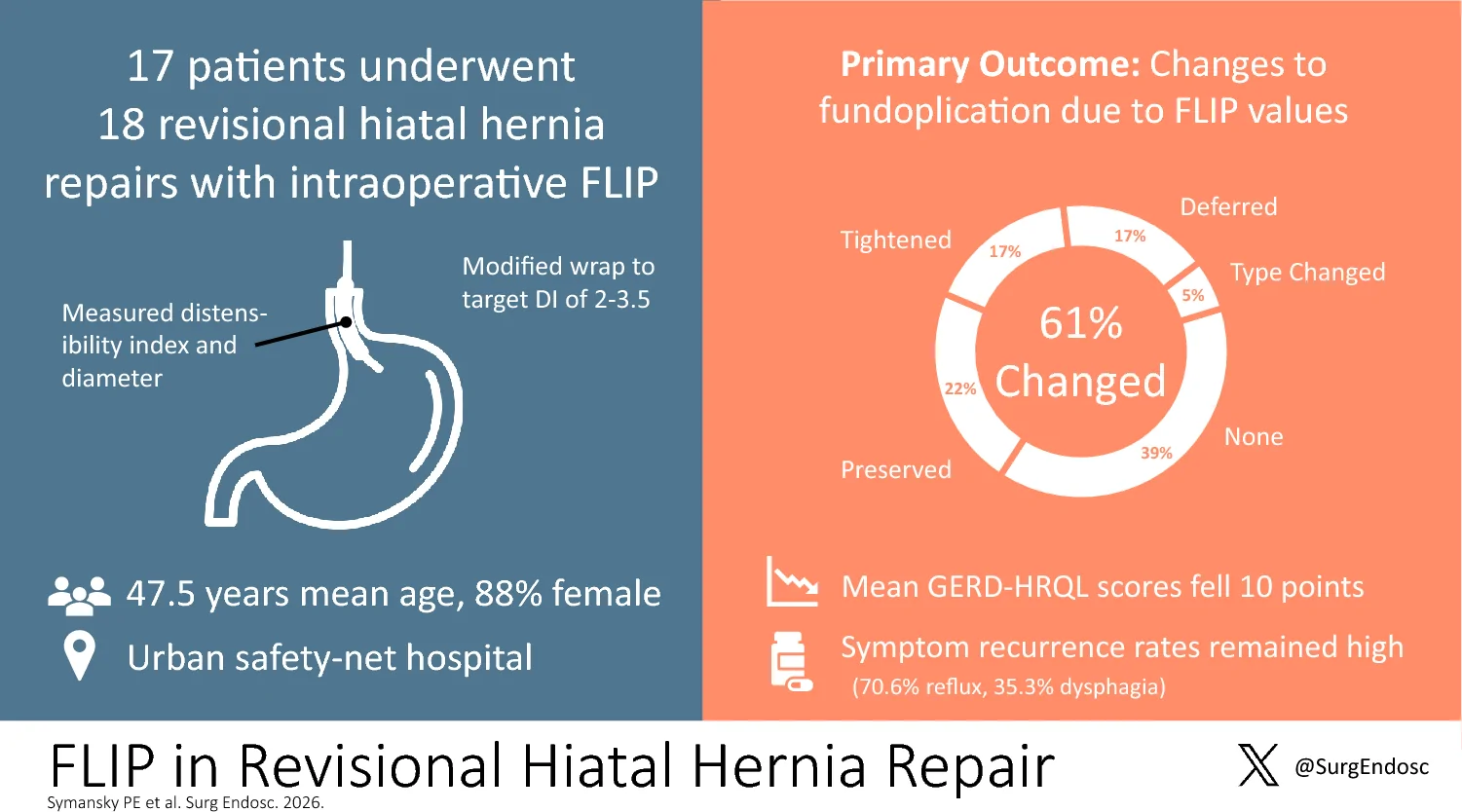

Despite advances in minimally invasive techniques, hiatal hernia repair (HHR) remains a technically demanding intervention, and long-term durability is an obstinate challenge. Recurrence rates following primary repair are substantial, ranging from 15% to over 50%, depending on how recurrence is defined and the length of follow-up [1–3]. Consequently, a significant number of patients require reoperation for recurrent anatomic failure or recurrent symptoms, such as severe heartburn, regurgitation, or dysphagia.

Revisional hiatal hernia repair (rHHR) is inherently more complex than index repair. The surgeon must navigate a “hostile hiatus” where dense adhesions obliterate normal anatomic planes, the esophagus is often shortened by chronic inflammation, and the gastric fundus may be compromised from prior mobilization or fundoplication. These factors contribute to rHHR being associated with higher morbidity, often from increased risk of perforation, and lower success rates when compared to primary intervention [4, 5]. Furthermore, determining the appropriate surgical strategy is challenging, especially if relying exclusively on visual inspection. As a result, revisional surgery traditionally involves certain steps that we hypothesize may not always be necessary, such as taking down and recreating the fundoplication.

Historically, bougie calibration, endoscopic inspection, or tactile feedback have been used to provide elements of objectivity to intraoperative assessment, but these are still largely subjective measures. These methods provide a static assessment of a dynamic sphincter and correlate poorly with postoperative physiologic function [6]. To bridge this gap, the functional luminal imaging probe (FLIP) was developed. FLIP utilizes impedance planimetry to provide real-time, quantitative data representing the geometric and mechanical properties of the esophagogastric junction (EGJ) [7].

While FLIP has demonstrated utility in optimizing primary anti-reflux surgery [8], its application in the revisional setting is less clear. The scarred, fibrotic tissues encountered in rHHR often exhibit altered compliance compared to virgin tissue. This study aims to evaluate the impact of intraoperative FLIP on surgical decision-making and assess long-term quality-of-life (QOL) outcomes in a cohort of complex patients undergoing rHHR at an urban safety-net hospital. We hypothesized that intraoperative FLIP values would guide management changes, facilitating a tailored approach that avoids unnecessary dissection while ensuring functional competence.

## Materials and methods

### Study design and population

**Fig. 1:**
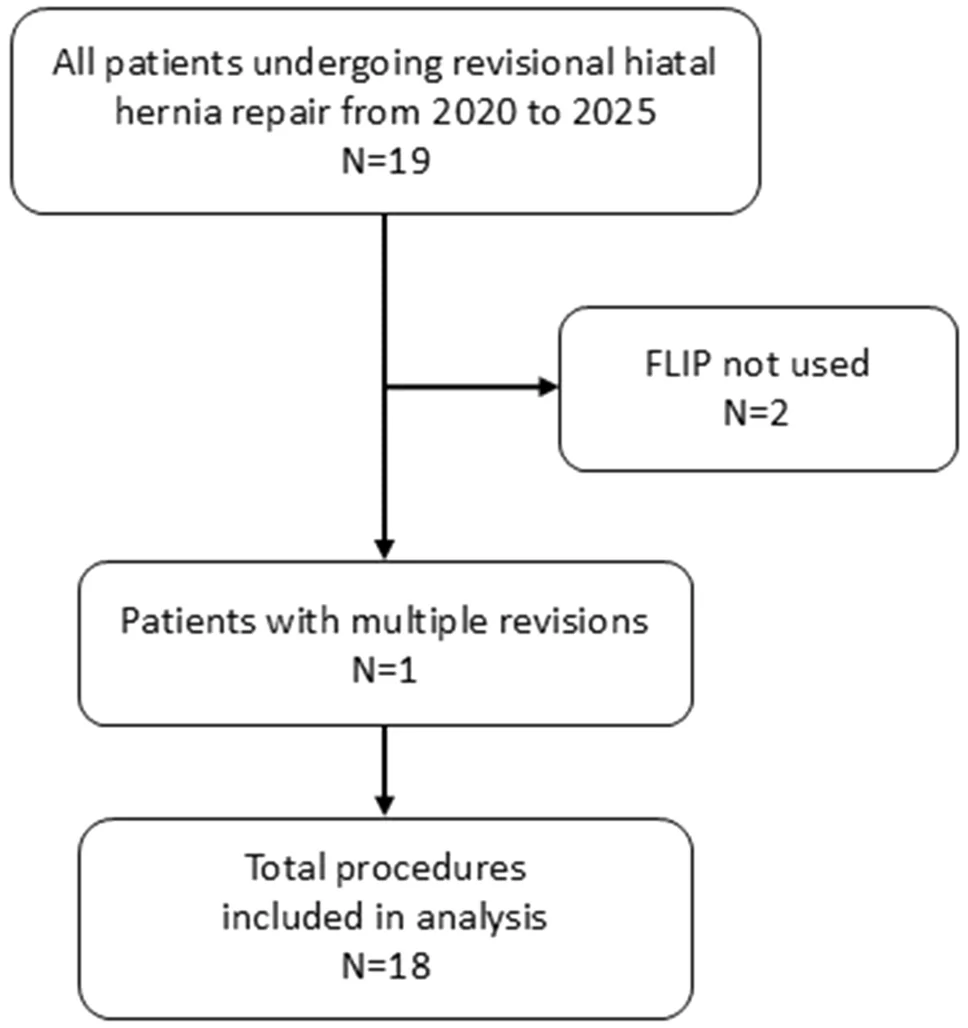
Inclusion criteria

This was an Institutional Review Board-approved retrospective review. The study population included all adult patients (age ≥ 18 years) undergoing revisional hiatal hernia repair between 2020 and 2025 at a high-volume, academic safety-net hospital. “Revisional” was defined as any operation following a prior anti-reflux procedure or prior hiatal hernia repair. Recurrent hiatal hernia was defined as objective radiographic evidence of the esophagogastric junction or gastric fundus displaced above the diaphragmatic hiatus, as documented in an upper gastrointestinal (UGI) series report. Exclusion criteria included cases where FLIP data could not be acquired due to technical failure. Patients unable to provide follow-up data were excluded from the follow-up analysis; the final selection process and inclusion criteria are summarized in Fig. 1. The study adhered to STROBE guidelines for observational studies [9].

### Surgical technique

**Fig. 2:**
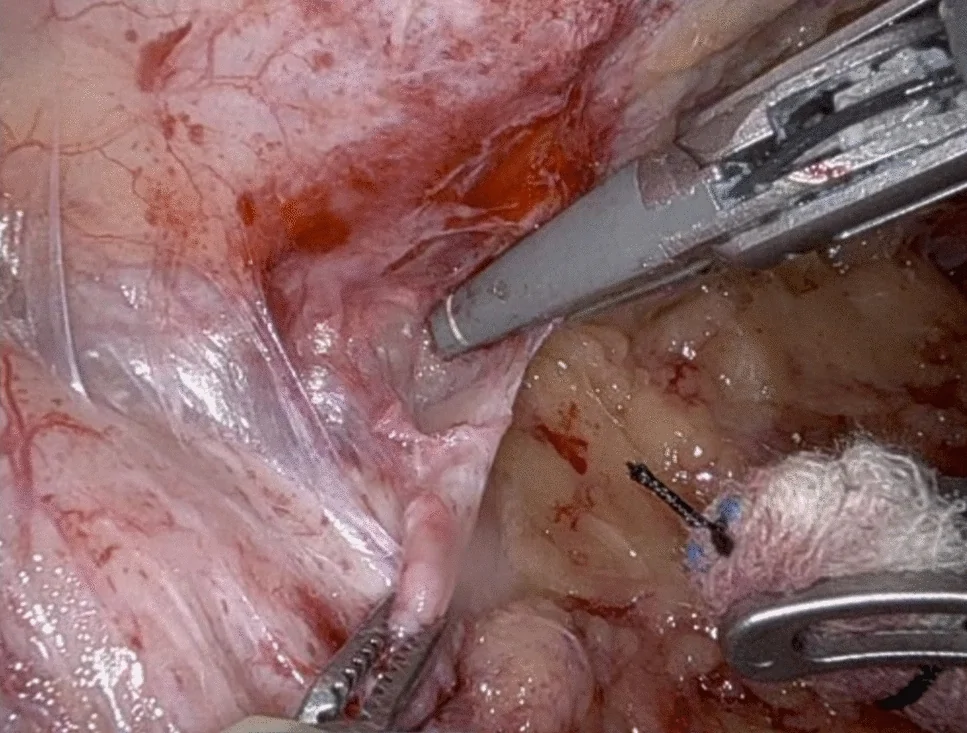
Dense adhesions encountered during revisional hiatal hernia repair

**Fig. 3:**
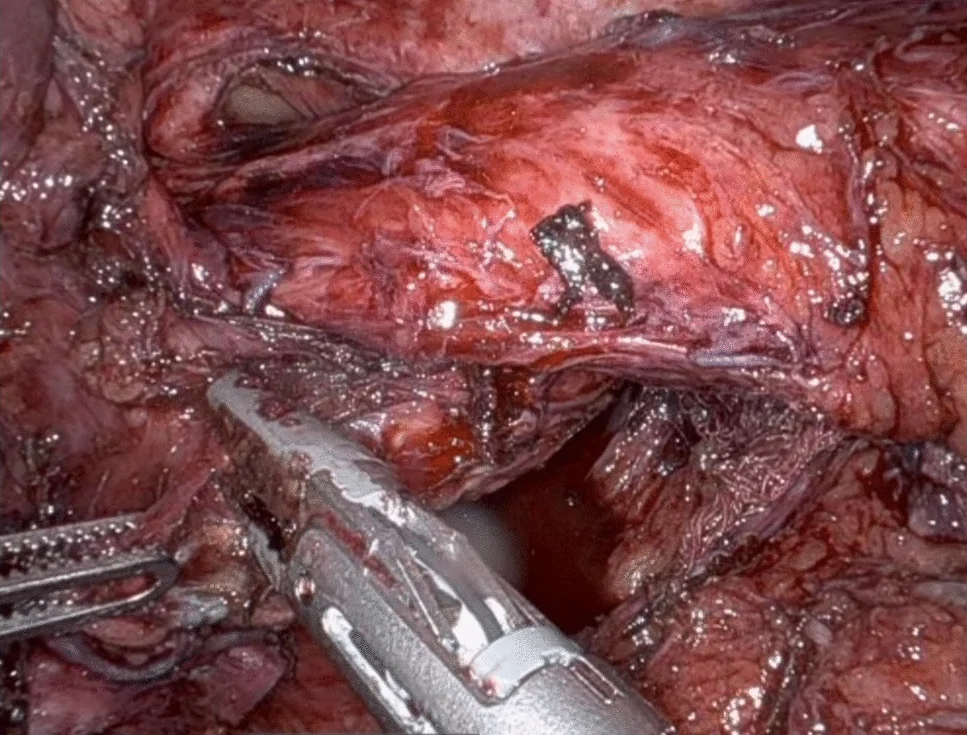
Reversing a fundoplication during revisional repair

All procedures were performed using a robot-assisted minimally invasive approach by a single surgeon (KE). Following abdominal access and adhesiolysis (Fig. 2), the hernia sac was reduced and excised. Great care was taken to identify and preserve the vagus nerves when possible. The previous fundoplication was then inspected transabdominally and endoscopically. If the wrap appeared intact and well-positioned, further dissection was deferred until intraoperative FLIP assessment. On the other hand, disrupted, slipped, or herniated wraps were completely taken down (Fig. 3). The crura were re-approximated posteriorly using non-absorbable sutures and a U-shaped bioabsorbable mesh (BIO-A® Tissue Reinforcement, GORE®, Newark, DE) was placed to reinforce the repair in all cases.

### Intraoperative FLIP protocol

The EndoFLIP™ impedance planimetry system (Medtronic Inc, Shoreview, MN) was used, with an EF-325 8-cm balloon catheter containing 16 independent sensors. A previously published expert consensus protocol was used [10, 11]. The patient was kept in reverse Trendelenburg position and pneumoperitoneum was released when FLIP data were obtained. Values included diameter (D), cross-sectional area (CSA), and distensibility index (DI), taken at three distinct timepoints: (1) post-dissection/reduction, (2) post-crural closure with mesh, and (3) post-fundoplication, if performed. Data were recorded at balloon fill volumes of 30 mL, 40 mL, and 50 mL, after approximately 30 s, once values stabilized. Values obtained at the 40 mL balloon fill volume were preferentially used to guide procedural changes, provided the balloon reached a minimum pressure of 15 mmHg, otherwise values obtained at the 50 mL fill volume were used.

**Fig. 4:**
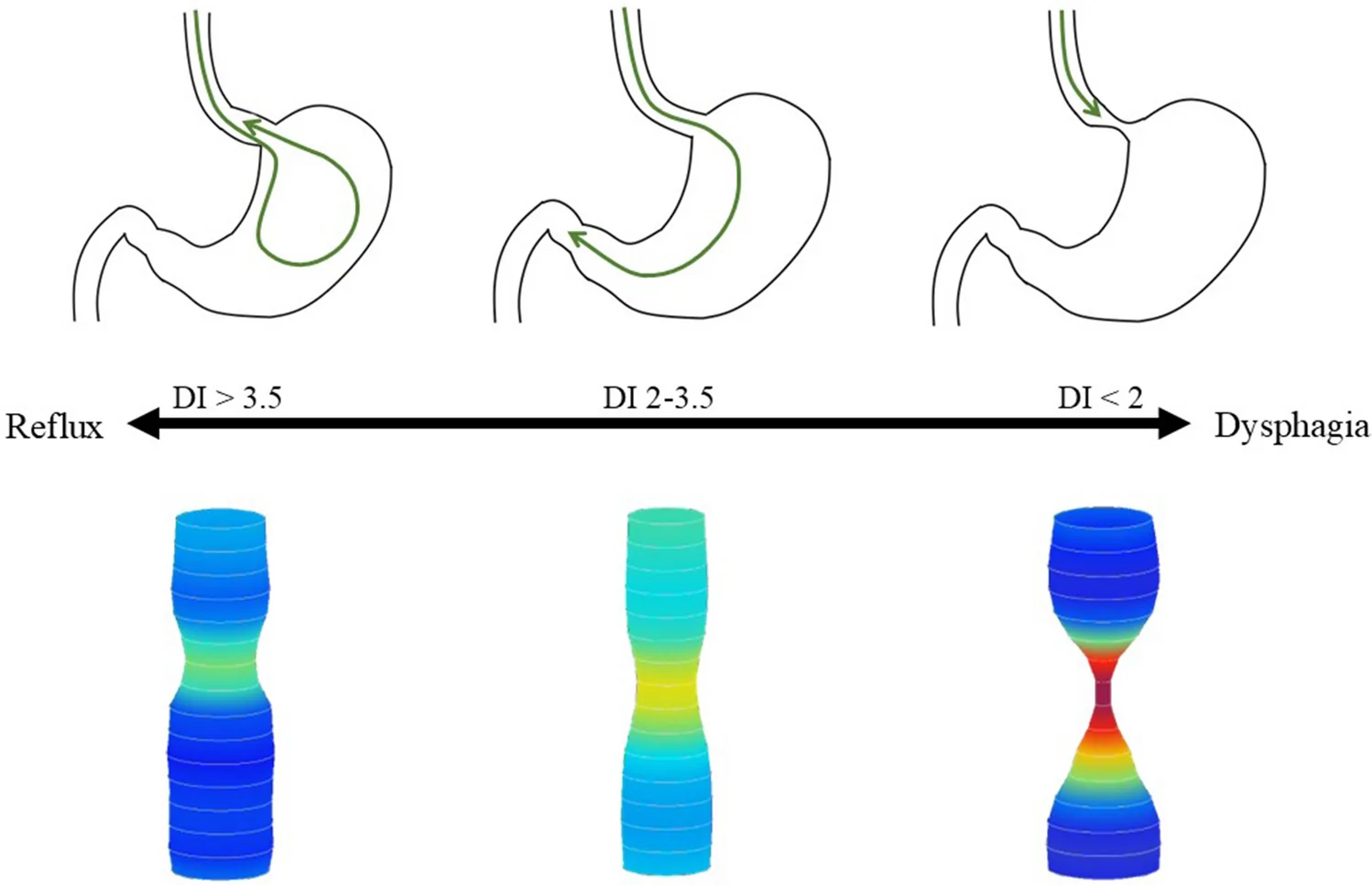
Interpretation of distensibility indices

Surgical decision-making followed a standardized algorithm targeting a DI of 2.0–3.5 mm^2^/mmHg to achieve appropriate anti-reflux outcomes, based on previously established normative values (Fig. 4) [8, 10].

- **DI < 2.0 mm**^**2**^**/mmHg:** Indicated a high-pressure zone with low compliance and high risk of dysphagia. Management involved loosening crural sutures, converting Nissen to Toupet, or deferring fundoplication.
- **DI > 3.5 mm**^**2**^**/mmHg:** Indicated a compliant, loose sphincter with high risk of reflux recurrence. Management involved tightening the crural closure, adding a fundoplication, or tightening an existing fundoplication.
- **DI 2—3.5 mm**^**2**^**/mmHg:** Indicated adequate anti-reflux control. If these values were achieved following crural closure in the presence of a pre-existing fundoplication, provided it appeared morphologically intact by both endoscopic and laparoscopic inspection, dismantling of the wrap was deferred.

### Outcomes and statistical analysis

The primary outcome was a measurement of intraoperative management changes guided by FLIP. Secondary outcomes were quality-of-life metrics and patient-reported symptoms measured with the GERD-HRQL score (range 0–50) until last clinical follow-up. Statistical analysis was performed using descriptive statistics. Continuous variables were summarized as means ± standard deviations (SD). Preoperative and postoperative GERD-HRQL scores were compared using a two-tailed unpaired *t* test. A *p* value of < 0.05 was considered statistically significant.

## Results

### Demographics and operative characteristics

A total of 17 patients who underwent 18 revisional operations were identified (Table 1). One patient required a second revisional surgery, which was included in the analysis as a separate procedure, due to an early failure at 8.7 months after the patient’s first revision. The cohort was predominantly female (88%) with a mean age of 47.5 ± 14.1 years. The primary indication for revision was recurrent hernia or reflux in 16 patients (94%), while 1 patient (6%) presented with primary dysphagia or obstruction. Type I (sliding) and Type III (mixed) hernias were the most common anatomic presentations. Most patients did not undergo preoperative esophageal manometry (Table 2), but of those who did, 3 patients showed evidence of a hypotensive lower esophageal sphincter, and 3 patients showed evidence of ineffective motility.

**Table 1:** Baseline demographics

Characteristic	Value (*n* = 17)
Mean age	47.5 ± 14.1 years
Gender (*n*, %)	
Female	15 (88%)
Male	2 (12%)
Prior surgical history	
1 prior repair	14 (82%)
>1 prior repair	3 (18%)
Prior fundoplication	15 (88%)
Primary indication for revision	
Recurrent hernia/reflux	16 (94%)
Dysphagia/obstruction	1 (6%)
Hiatal hernia type (*n*, %)	
Type I	5 (29%)
Type II	1 (6%)
Type III	10 (59%)
Type IV	1 (6%)

**Table 2:** Preoperative esophageal manometry

	Value (*n* = 17)
Underwent manometry (*n*, %)	8 (47%)
Results	
Normal (*n*, %)	2 (12%)
Hypotensive LES (*n*, %)	3 (18%)
Ineffective motility (*n*, %)	3 (18%)

### Intraoperative FLIP data and management changes

**Fig. 5:**
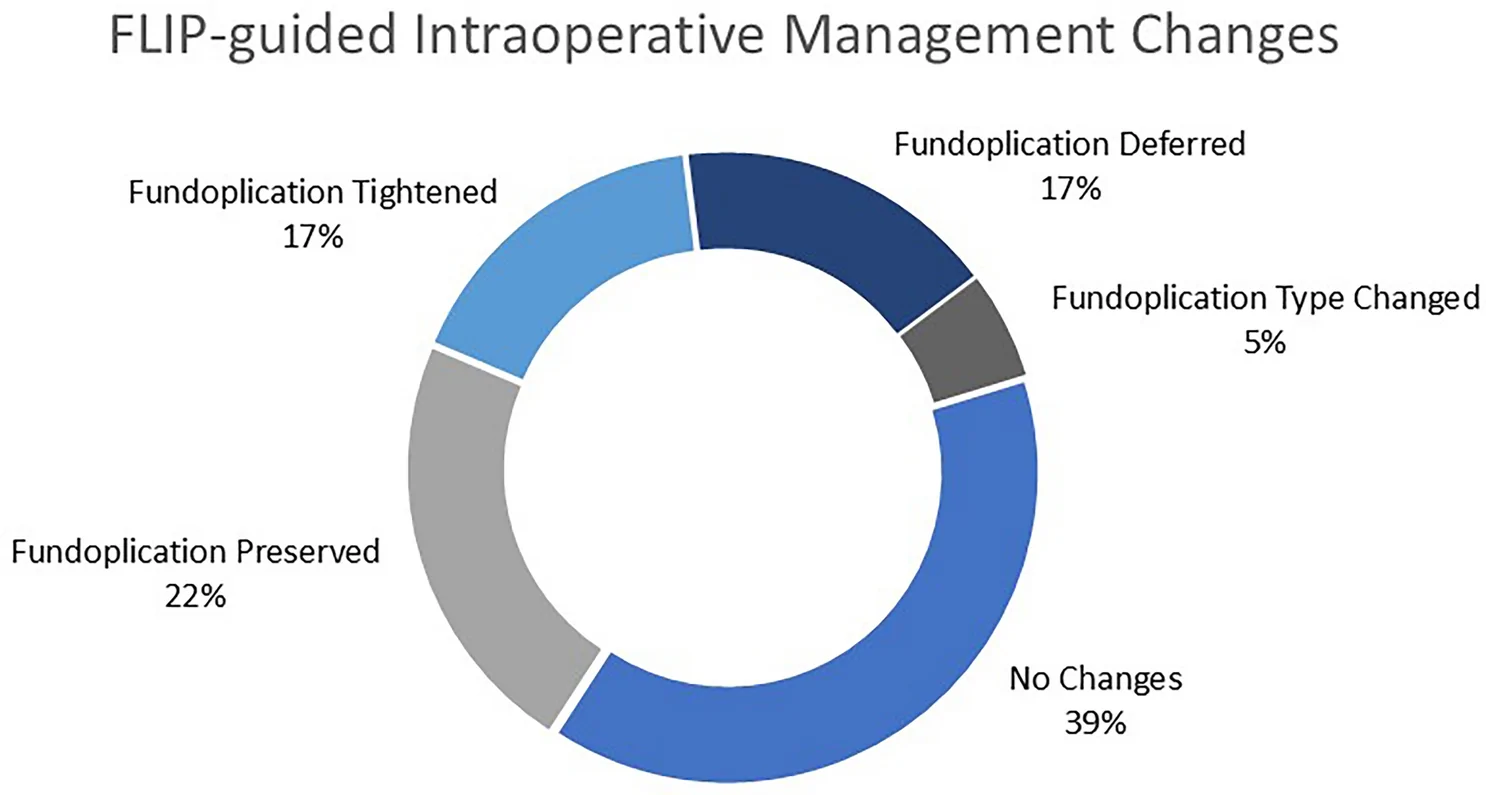
Management changes based on intraoperative FLIP values

Intraoperative FLIP values influenced surgical decision-making in 61% (11/18) of cases. Mean distensibility indices after cruroplasty and fundoplication were 2.60 and 1.93, respectively, obtained at the 40 mL balloon fill volume (Table 3). Consistent with previously published data, the greatest decrease in DI occurred after cruroplasty, and not after fundoplication [12, 13]. The specific alterations to the operative plan are detailed in Fig. 5.

- **Preservation of Fundoplication (36%):** In four cases, FLIP demonstrated appropriate distensibility, indicating that the pre-existing anti-reflux mechanism remained intact. Dissection of the fundus was deferred, and only reduction of the fundoplication and hiatal defect was performed.
- **Deferral of Fundoplication (27%):** In three cases, a deferral of fundoplication occurred where a pre-existing wrap was taken down but not reconstructed, as ideal distensibility had been achieved. Fundoplication was omitted to prevent postoperative dysphagia.
- **Tightening of Fundoplication (27%):** In three cases, FLIP indicated high compliance concerning for postoperative reflux, prompting the addition of sutures or tightening the wrap.
- **Change in Fundoplication Type (9%):** In one case, the planned fundoplication type was converted (Nissen to Toupet) to achieve the target DI. This change was necessitated by the discovery of a disrupted existing Nissen and guided by FLIP data to achieve optimal distensibility.

**Table 3:** Mean FLIP distensibility indices (DI) and diameters (D) at 30 mL, 40 mL, and 50 mL balloon fill volumes

	D_30_	DI_30_	D_40_	DI_40_	D_50_	DI_50_
Post hiatal dissection	12.88	6.38	13.79	5.07	15.59	4.06
Post cruroplasty	9.10	3.68	12.54	2.60	12.36	2.08
Post fundoplication	10.45	3.40	10.00	1.93	11.54	1.57
Delta, hiatal dissection to cruroplasty		−4.40		−2.79		−2.27
Delta, cruroplasty to fundoplication		−1.30		−0.60		−0.41

Among patients who ultimately had a fundoplication (new or preserved), 64% received a Toupet (270° or less) and 36% received a Nissen (360°). Bioabsorbable mesh was used in 100% of cases. The median length of stay was 2 days (range 1–10).

### Clinical outcomes

Quality-of-life data were available with a mean follow-up of 24.9 months. The mean GERD-HRQL score improved (Table 4) from 26.7 ± 8.5 preoperatively to 16.5 ± 12.9 postoperatively (*p* = 0.034).

**Table 4:** Preoperative versus postoperative GERD-HRQL scores (*n* = 17)

	Preoperative	Postoperative	*p*-value
GERD-HRQL score (Mean ± SD)	26.7 ± 8.5	16.5 ± 12.9	0.034

Following our institutional protocol, patients routinely underwent an upper gastrointestinal (UGI) series at a mean of 14.3 months (range, 0.4–54.1) postoperatively. Radiographic follow-up was available for 16 patients. Within this group, radiographic recurrence was identified in 7 patients (43.8%), defined as objective evidence of the esophagogastric junction or gastric fundus displaced above the diaphragmatic hiatus. Among the subset of recurrences with documented measurements (*n* = 3), the mean size of the hiatal defect was 3.9 cm (range, 3.5–4.1 cm); all four remaining recurrences were qualitatively described as small type 1 hiatal hernias, and no large recurrences were observed in the study cohort.

**Fig. 6:**
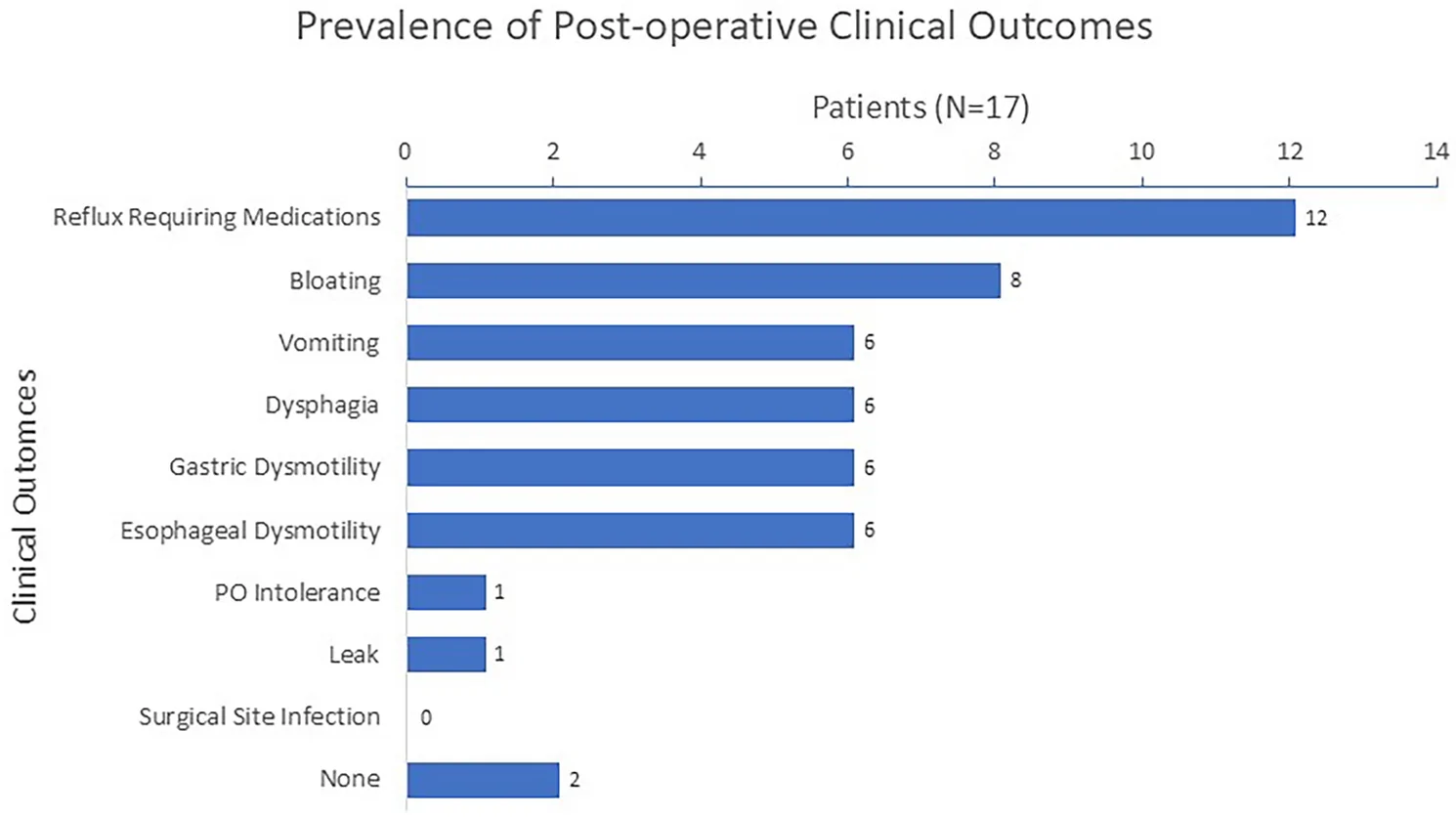
Prevalence of postoperative clinical outcomes following revisional hiatal hernia repair. Values represent the percentage of the total cohort (*n* = 17) reporting each symptom or complication at a mean follow-up of 38.8 months

At a mean follow-up of 38.8 months, the cohort demonstrated a diverse profile of functional and technical outcomes (Fig. 6). The most prevalent functional symptoms included recurrent reflux requiring medical management, affecting 12 patients (70.6%), and abdominal bloating, affecting 8 patients (47.1%). A subset of 6 patients (35.3%) experienced a cluster of symptoms including dysphagia, vomiting, and both gastric and esophageal dysmotility. Despite this overall symptom burden, 2 patients (11.8%) remained completely asymptomatic.

The incidence of major surgical complications was low (Table 5). One patient, who required a Collis-type gastrectomy for esophageal lengthening, experienced a staple line leak on postoperative day 9. This was managed with a return to the operating room for laparoscopic washout, gastrostomy tube placement, and endoscopic placement of a partially covered esophageal stent. There were no reported incidences of surgical site infection (0.0%). While many patients reported persistent symptoms, 13 patients (76.5%) were successfully managed nonoperatively. Four patients (23.5%) required further surgical intervention for indications including recurrent hernia and wrap migration at a mean of 11.9 months (range, 7.9–17.0) following the index procedure.

**Table 5:** Postoperative complications

	Value (*n* = 17)
Surgical site infection	0 (0%)
Reoperation for persistent symptoms	4 (23.5%)
Mean interval before reoperation, in months	11.9

## Discussion

In this small cohort of rHHR cases, real-time intraoperative FLIP data prompted changes in surgical management in 61% of cases, challenging the traditional paradigm of reoperative hiatal hernia repair, which typically involves complete takedown of the prior wrap, hiatal closure, and construction of a new fundoplication. This rate of management modification exceeds that reported in primary hiatal hernia cohorts, where FLIP altered intraoperative decision-making in approximately 39% of cases, underscoring its potentially greater utility in the revisional setting where anatomy and physiology are more heterogeneous [8].

In our study, one-third of patients demonstrated preservation of the anti-reflux function of the wrap despite hernia recurrence. Avoiding wrap takedown may shorten operative time, reduce technical complexity, and mitigate surgical risk. These findings align with prior work demonstrating that FLIP can lead to deferral or modification of fundoplication when adequate anti-reflux physiology is achieved after hiatal closure alone, highlighting the limitations of a “one-size-fits-all” reconstructive approach [8, 14]. Importantly, prior studies have shown that the greatest reduction in distensibility occurs after hiatal closure, with comparatively smaller changes after fundoplication, suggesting that restoration of crural integrity may be the dominant contributor to EGJ competence in select patients [8, 12, 15].

FLIP technology also enabled identification of patients with a stiffened esophagus, in whom any fundoplication would likely result in dysphagia. In these cases, deferring fundoplication prioritized swallowing function over complete reflux control. This individualized, physiology-driven approach is further supported by emerging data demonstrating that intraoperative FLIP measurements correlate with postoperative outcomes and can be used to define an optimal “therapeutic window” of EGJ distensibility, balancing reflux control against the risk of dysphagia [14, 16].

Our outcomes should be interpreted in the context of a safety-net population with a high burden of comorbidities, including gastroparesis and esophageal dysmotility [17, 18]. The rate of persistent symptoms at long-term follow-up likely reflects these underlying motility disorders rather than anatomic failure of the repair. Notably, despite persistent symptoms, nearly two-thirds of patients were managed nonoperatively, suggesting that persistent symptoms may be modulated without reintervention when anatomy is appropriately restored. These findings are consistent with the broader literature, demonstrating that while fundoplication reliably improves objective reflux parameters, symptom outcomes are influenced by underlying esophageal function and patient-specific factors [19].

Of note, the use of bioabsorbable mesh in all cases was not associated with mesh-related complications, supporting its short-term safety profile in the revisional setting [20, 21].

This study is limited by its retrospective design, small sample size, and lack of control group, which prevents making definitive conclusions regarding the impact of intraoperative FLIP. The absence of a control group reflects the early adoption of this technology at our institution, during which very few patients underwent revisional hiatal hernia repair without it. Additionally, patient comorbidities were not included in the analysis, and the absence of preoperative FLIP data precludes assessment of changes in esophageal compliance at the individual level. Furthermore, preoperative esophageal manometry was not routinely obtained, which is a result of the heterogeneity of symptoms in patients undergoing revisional hiatal hernia repair. Many of these patients presented with indications for reoperation other than strictly reflux, for which manometric data would not have changed surgical management. Importantly, FLIP is not intended to replace manometry, although emerging evidence suggests FLIP may serve as an imperfect surrogate in select patients who cannot tolerate manometry [22–24].

The variability in postoperative outcomes highlights the inherent complexity of revisional foregut surgery, where outcomes are generally less favorable and more heterogeneous than those reported after primary hiatal hernia repair [25–30]. Despite these limitations, this study represents, to our knowledge, the largest reported series evaluating intraoperative FLIP specifically in revisional hiatal hernia repair. The high rate of intraoperative management modification, coupled with growing evidence supporting FLIP-guided tailoring of foregut procedures, suggests that this technology may be particularly valuable in navigating the complexity of reoperative surgery. These findings provide important preliminary data to support larger, multicenter comparative studies that can better define the efficacy and optimal therapeutic target values for FLIP in revisional foregut surgery, particularly as scarring and local tissue changes may alter measurements compared with primary hiatal hernia repair.

In conclusion, intraoperative FLIP provides real-time physiologic data that frequently alters surgical management in revisional hiatal hernia repair. This technology could help guide more tailored, patient-specific revisional strategies balancing reflux control with preservation of esophageal function. These findings support FLIP as a valuable adjunct in reoperative foregut surgery, warranting further study in larger cohorts.
